# Transgenerational modification of dopaminergic dysfunctions induced by maternal immune activation

**DOI:** 10.1038/s41386-020-00855-w

**Published:** 2020-09-12

**Authors:** Ulrike Weber-Stadlbauer, Juliet Richetto, Ramona A. J. Zwamborn, Roderick C. Slieker, Urs Meyer

**Affiliations:** 1 Institute of Pharmacology and Toxicology, University of Zurich-Vetsuisse, Zurich, Switzerland; 2 Neuroscience Center Zurich, University of Zurich and ETH Zurich, Zurich, Switzerland; 3 Department of Neurology, UMC Utrecht Brain Center, Utrecht, The Netherlands; 4 Department of Biomedical Data Sciences, Section Molecular Epidemiology, Leiden, The Netherlands; 5 Department of Cell and Chemical Biology, Leiden University Medical Center, Leiden, The Netherlands

**Keywords:** Neuroimmunology, Developmental disorders

## Abstract

Prenatal exposure to infectious and/or inflammatory insults is increasingly recognized to contribute to the etiology of psychiatric disorders with neurodevelopmental components. Recent research using animal models suggests that maternal immune activation (MIA) can induce transgenerational effects on brain and behavior, possibly through epigenetic mechanisms. Using a mouse model of MIA that is based on gestational treatment with the viral mimeticpoly(I:C) (= *polyriboinosinic-polyribocytidilic* acid), the present study explored whether the transgenerational effects of MIA are extendable to dopaminergic dysfunctions. We show that the direct descendants born to poly(I:C)-treated mothers display signs of hyperdopaminergia, as manifested by a potentiated sensitivity to the locomotor-stimulating effects of amphetamine (Amph) and increased expression of tyrosine hydroxylase (*Th*) in the adult ventral midbrain. In stark contrast, second- and third-generation offspring of MIA-exposed ancestors displayed blunted locomotor responses to Amph and reduced expression of *Th*. Furthermore, we found increased DNA methylation at the promoter region of the dopamine-specifying factor, nuclear receptor-related 1 protein (*Nurr1*), in the sperm of first-generation MIA offspring and in the ventral midbrain of second-generation offspring of MIA-exposed ancestors. The latter effect was further accompanied by reduced mRNA levels of Nurr1 in this brain region. Together, our results suggest that MIA has the potential to modify dopaminergic functions across multiple generations with opposite effects in the direct descendants and their progeny. The presence of altered DNA methylation in the sperm of MIA-exposed offspring highlights the possibility that epigenetic processes in the male germline play a role in the transgenerational effects of MIA.

## Introduction

Infectious or noninfectious maternal immune activation (MIA) is an environmental risk factor of psychiatric and neurological disorders with neurodevelopmental etiologies [1]. This epidemiological association also receives strong support from experimental work in animal models, which have identified a broad spectrum of behavioral, physiological, and molecular alterations in offspring of MIA-exposed mothers [2–4]. In addition to the neurodevelopmental disturbances emerging in the direct descendants of exposed mothers, MIA further induces transgenerational effects on brain and behavior [5, 6]. Thus far, evidence for the latter effects has been obtained from mouse models, in which MIA was induced by the viral mimeticpoly(I:C) [7–9], or the bacterial endotoxin lipopolysaccharide [10]. Using the poly(I:C)-based mouse model, we previously found transgenerational effects of MIA on social and fear-related behaviors, which were transmitted via the paternal lineage and were stable until the third generation [9]. These findings are consistent with the concept of transgenerational nongenetic inheritance of pathological traits [11] and suggest that epigenetic modifications in (male) gametes may contribute to, or even mediate, the transgenerational effects of MIA.

In the present study, we investigated whether the transgenerational effects of MIA are extendable to dysfunctions in the central dopamine (DA) system. While MIA has the potential to modify various neurotransmitter systems [1–6], our primary motivation to focus on the DA system was twofold. First, abnormal functioning of the DA system is a pathological hallmark of various psychiatric disorders, including schizophrenia and related psychotic disorders [12], bipolar disorder [13], and depressive disorders [14]. Second, functional abnormalities in the DA system have been widely documented in various rodent models of MIA [15–20], with a recent extension to a nonhuman primate model of MIA [21]. It remains unknown, however, whether MIA-induced changes in DA functions can be transmitted across generations. Exploring the latter provided the main impetus of the current study.

Based on the existing evidence, there is a genuine possibility for transgenerational effects on DA functions to occur after MIA. Indeed, gestational exposure to psychological stressors, which induce placental inflammation and behavioral anomalies in the offspring [22], were previously found to induce transgenerational effects on DA functions in mice, including altered DNA methylation of dopaminergic genes [23, 24]. Similar transgenerational effects have also been identified in animal models of maternal overnutrition [25], which is typically accompanied by obesity-induced inflammation [26]. Finally, it was recently shown that infection of male mice with the parasite *Toxoplasma gondii* (*T. gondii*) alters epigenetic profiles of sperm cells and causes transgenerational behavioral changes [27]. Even if this recent study did not specifically explore transgenerational effects of *T. gondii* infection on DA functions, it provides support to the hypothesis that antenatal immune challenges can induce transgenerational effects via stable epigenetic modifications in male gametes.

Here, we used the poly(I:C)-based mouse model [4] to study the transgenerational effects of MIA on DA dysfunctions. Functional changes in the DA system were assessed by testing the animals’ locomotor response to the indirect DA receptor agonist, amphetamine (Amph) [20, 28]. Transcriptional profiling of tyrosine hydroxylase (*Th*) and nuclear receptor-related 1 protein (*Nurr1*; also known as *Nr4a2*) was conducted to explore whether MIA induces transgenerational effects on DA-related gene transcription in the ventral midbrain (vMB), which contains the majority of post-mitotic DA cells in the mammalian brain [29]. Whereas *Th* is the rate-limiting enzyme for DA synthesis [30], *Nurr1* is an obligatory transcription factor for midbrain DA cell development [31] and exerts a number of molecular functions in post-mitotic midbrain DA neurons, including the regulation of *Th* [32, 33].

In addition to quantifying gene expression of *Th* and *Nurr1*, we assessed the levels of DNA methylation in their promoter regions. DNA methylation is one of various epigenetic processes, which together confer short- and long-term changes in gene expression without altering the DNA code itself [11, 34]. States of DNA methylation can be maintained during cell division, and therefore, they may provide a molecular mechanism for the propagation of heritable changes in gene expression and for transgenerational non-genetic inheritance of environmentally acquired traits [11, 34]. Here, we measured DNA methylation of *Th* and *Nurr1* gene promoters in the vMB to test the hypothesis that the anticipated dysregulation of *Th* and *Nurr1* transcription in the vMB of MIA-exposed offspring would be associated with altered DNA methylation in the corresponding gene promoters [35]. Furthermore, we assessed DNA methylation of *Th* and *Nurr1* promoter regions in the sperm of MIA-exposed and control offspring in order to evaluate whether epigenetic modifications in male gametes may provide a molecular mechanism by which the effects of MIA can be transmitted from one generation to the next.

## Materials and methods

### Animals

C57BL6/N mice (Charles River, Sulzfeld, Germany) were used throughout the study. The animals were kept under a reversed light–dark cycle (lights off: 7:00 A.M. to 7:00 P.M.) as described before [9] and in the Supplementary information. All procedures described in the present study had been previously approved by the Cantonal Veterinarian’s Office of Zurich, and all efforts were made to minimize the number of animals used and their suffering.

### Timed mating and maternal immune activation in F0 mothers

A reporting guideline checklist for the MIA model [36] is provided in Supplementary Table S1. To generate the first-generation (F1) offspring of poly(I:C)-exposed or control mothers, female mice were subjected to a timed-mating procedure as established before [9] (for details, see the Supplementary Information). Pregnant F0 dams on gestation day (GD) 9 were randomly assigned to receiving either a single injection of poly(I:C) (potassium salt, P9582, lot number: 086M4045; Sigma–Aldrich, Switzerland) or vehicle (sterile pyrogen-free 0.9% NaCl) as described before [9] and in the Supplementary information. For experimental series involving F0 exposures, a total of 18–24 pregnant dams were used. Maternal behavior was assessed for every generation (see Supplementary Information and Supplementary Fig. S1).

### Allocation of F1 offspring and production of subsequent generations

**Fig. 1:**
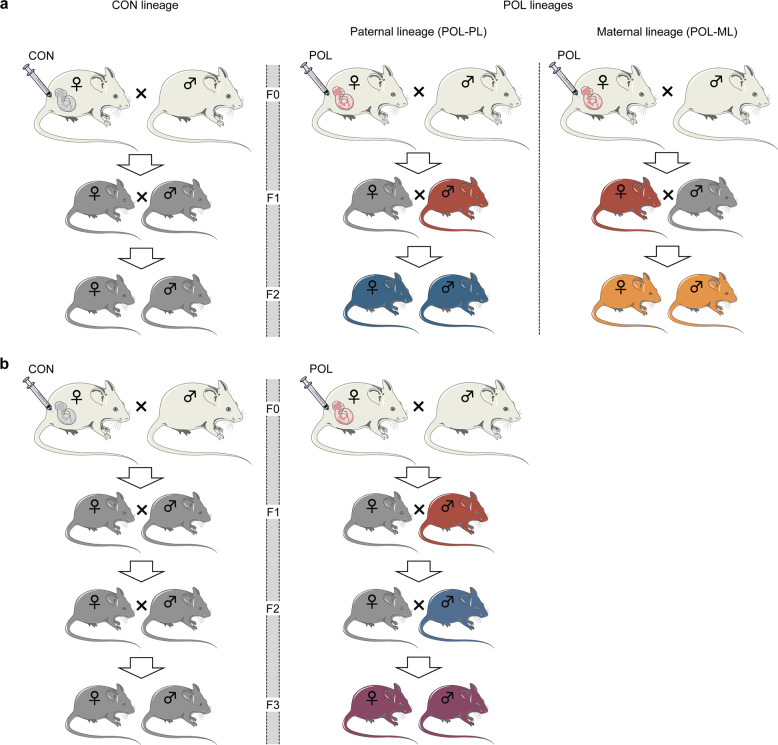
Breeding scheme used to generate F1, F2, and F3 offspring with control and poly(I:C)-derived ancestors. **a** Breeding scheme used to generate F1 and F2 generations with control and immune-challenged ancestors. Pregnant F0 mice were treated with vehicle control (CON) or poly(I:C) (POL) solution to obtain F1 CON and F1 POL offspring. To obtain F2 POL offspring via the paternal lineage (POL-PL), male F1 POL offspring were mated with female F1 CON offspring; and to generate F2 POL offspring via the maternal lineage (ML-POL), female F1 POL offspring were crossed with male F1 CON offspring. F1 CON males and females were crossed to obtain the F2 control lineage (F2 CON). **b** Breeding scheme used to obtain the F3 generation with control and immune-challenged ancestors deriving from the paternal lineage (PL). Pregnant F0 mice were treated with control (CON) or poly(I:C) (POL) solution, and F1 POL males were mated with F1 CON females to generate F2 POL-PL offspring. F2 POL-PL males were then mated with F2 CON females to obtain F3 POL-PL offspring. CON F3 offspring were generated by crossing F2 CON males and females.

All offspring were weaned on postnatal day (PND) 21 and littermates of the same sex were maintained in groups of 3–5 animals per cage. Upon reaching adulthood (PND 90 onwards), F1 offspring were either allocated to behavioral testing (see below), molecular analyses or breeding, the latter of which served to produce subsequent generations of immune-challenged or control ancestors. Hence, we always used behaviorally naïve littermates for molecular analyses and as breeding pairs to obtain the F2 and F3 generations, thereby avoiding possible confounds arising from prior behavioral testing. Timed-mating procedures, as described in the Supplementary Information, were used to generate F2 and F3 offspring. In one series of experiments, we dissected the maternal (ML) and paternal lineages (PL) of F1 poly(I:C) offspring for the subsequent generation of F2 offspring (Fig. 1a). In another series of experiments, we generated F3 offspring with poly(I:C)-exposed or control ancestors, thereby focusing on the PL (Fig. 1b), as fully described in the Supplementary Information. A summary of animals used in each experiment is provided in Table 1.

**Table 1:** The table specifies the number of offspring used in each experimental procedure.

Experiment		Number of offspring per litter, group (CON or POL) and generation (F1–F3)		
		F1	F2	F3
Amph sensitivity (Fig. 2)	*Amph*	N (CON) = 12 (7m, 5f) [10] N (POL) = 11 (6m, 5f) [11]	N (CON) = 5m [5] N (POL-PL) = 5m [5] N (POL–ML) = 5m [5]	N (CON) = 9m [8] N (POL-PL) = 9m [9]
	*Sal*	N (CON) = 11 (6m, 5f) [10] N (POL) = 11 (6m, 5f) [11]	N (CON) = 5m [5] N (POL-PL) = 5m [5] N (POL-ML) = 5m [5]	N (CON) = 7m [7] N (POL-PL) = 7m [7]
Gene expression (Fig. 3)		N (CON) = 7m [7] N (POL) = 7m [7]	N (CON) = 9m [7] N (POL-PL) = 7m [7]	N(CON) = 7m [6] N (POL-PL) = 7m [6]
DNA methylation (Fig. 4)	*Brain*	N (CON) = 9m [9] N (POL) = 9m [9]	N (CON) = 8 m [7] N (POL-PL) = 8 m [7]	n/a
	*Sperm*	N (CON) = 10m [10] N (POL) = 11m [10]	n/a	n/a

The number in square brackets denotes the number of litters, from which the animals were obtained. f, females; m, males.

### Amphetamine sensitivity test in F1, F2, and F3 offspring

**Fig. 2:**
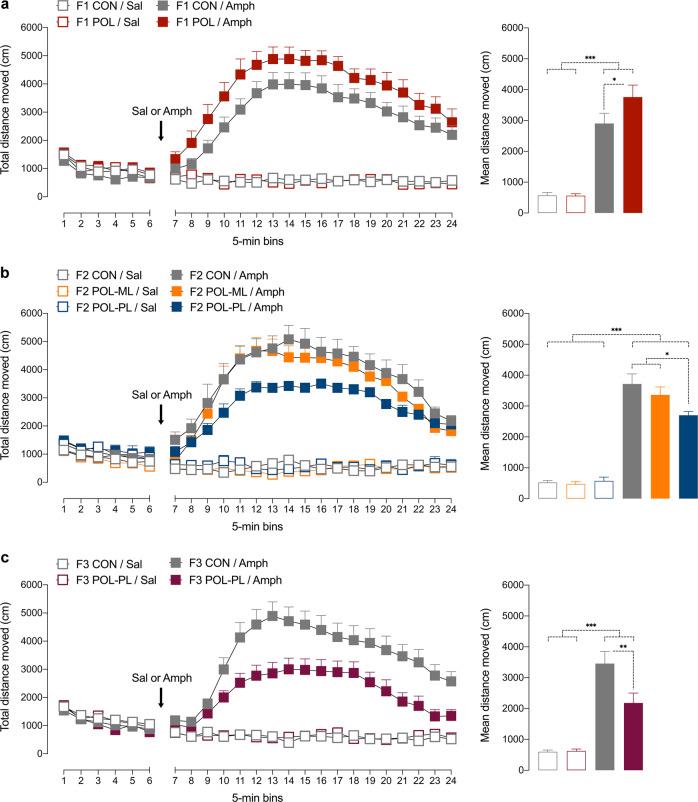
Effects of maternal immune activation on the locomotor reaction to acute systemic amphetamine challenge in F1, F2, and F3 offspring. All animals were first placed into the open field and allowed to habituate for 30 min, after which they were injected with amphetamine (Amph; 2.5 mg/kg) or saline (Sal) solution. The line plots show the distance moved as a function of 5-min-bins during the initial habituation and subsequent drug phase; the bar plots depict the mean distance moved during the drug phase. **a** Locomotor activity levels in F1 offspring of control (CON) and poly(I:C)-treated (POL) mothers. The data represent male and female offspring together, as there were no sex-specific effects of MIA on Amph sensitivity in F1 offspring. **b** Locomotor activity levels in male F2 CON offspring and male F2 POL offspring generated via the maternal (POL-ML) or paternal (POL-PL) lineages. **c** Locomotor activity levels in male F3 CON offspring and male F3 POL offspring generated via the paternal lineage (POL-PL). *p < 0.05, **p < 0.01, and ***p < 0.001, based on post-hoc comparisons. All values are means + SEM. The numbers of animals in each group are provided in Table 1.

For each generation, behavioral testing started when the offspring reached PND 90, where we assessed the animals’ sensitivity to Amph by measuring their locomotor activity in an open field [28, 37] (for details, see Supplementary Information). To minimize possible confounds arising from litter effects [38], 1–2 offspring per sex and litter were randomly selected and tested in the F1 generation, whereas 1–2 males per litter were selected and tested in the F2 and F3 generations. Hence, both male and female offspring were used in the first experimental series (Fig. 2a), whereas behavioral testing in the F2 and F3 generations were conducted using male offspring only. Based on our previous studies [9], a sample size of 9–16 offspring per group and sex (Table 1) was selected for the Amph sensitivity test. The Amph sensitivity test in F1-generation animals was replicated using an independent cohort of male and female poly(I:C)-exposed and control offspring (see Supplementary Fig. S2).

### Molecular analyses

RNA and DNA was isolated from the vMB and/or sperm of behaviorally naïve F1, F2, and F3 offspring of MIA-exposed ancestors and corresponding controls (Fig. 1) as described in the Supplementary Information. Messenger RNA (mRNA) and DNA methylation differences were quantified by real-time PCR analysis and EpiTYPER, respectively, as described in the Supplementary Information.

### Statistical analyses

All data were analyzed by parametric analysis of variance (ANOVA) followed by Tukey’s post-hoc test for multiple comparisons whenever appropriate, as described in the Supplementary Information. Our primary statistical approach used the individual offspring as the experimental unit, whereas our secondary statistical approach considered the treated mothers (i.e., litters) as experimental unit. The outcomes of the primary statistical approach are reported in the main manuscript, whereas the statistical results obtained by the secondary approach are reported in the Supplementary Information (Supplementary Table S4–S6). Importantly, the two statistical approaches provided fully consistent results. Statistical analyses were performed using StatView (version 5.0) and Prism (version 8.4) and statistical significance was set at *p* < 0.05.

## Results

### Transgenerational modification of amphetamine sensitivity

To identify possible transgenerational effects of poly(I:C)-induced MIA on dopaminergic functions, we first compared the sensitivity to the locomotor-enhancing effects of an acute Amph challenge in F1, F2, and F3 offspring of control or poly(I:C)-exposed ancestors. The systemic administration of Amph (2.5 mg/kg, i.p.) resulted in a general increase in locomotor activity, which peaked at around 30 min post-injection. Compared to F1 controls, F1 offspring born to poly(I:C)-treated mothers displayed a significant increase in Amph-induced locomotor hyperactivity (Fig. 2a). ANOVA of distance moved in the open field yielded a significant interaction between prenatal treatment and drug treatment (*F*_(1,37)_ = 4.44, *p* < 0.05); and subsequent post-hoc comparisons confirmed the significant difference between control and MIA-exposed F1 offspring (*p* < 0.05) in terms of the mean distance moved after Amph treatment. The MIA-induced potentiation of Amph sensitivity in F1 offspring was not significantly influenced by sex, as there was no significant interaction between prenatal treatment and sex (*F*_(1,37)_ = 1.35, *p* = 0.25), or between prenatal treatment, drug treatment and sex (*F*_(1,37)_ = 0.48, *p* = 0.49). Furthermore, control and MIA-exposed F1 offspring did not differ in terms of spontaneous locomotor activity (as measured during the initial habituation phase of the test) and with regards to the locomotor response to Sal administration (Fig. 2a).

Contrary to the effects in F1 offspring, second-generation (F2) offspring of MIA ancestors displayed a significant decrease in Amph-induced locomotor hyperactivity when compared to F2 control offspring. However, this decrease only emerged when F2 offspring of MIA-exposed ancestors were derived from the paternal lineage (Fig. 2b). F2 offspring of MIA-exposed ancestors did not display altered locomotor reaction to Amph when they stemmed from the maternal MIA ancestor lineage (Fig. 2b). ANOVA of distance traveled in the open field revealed a significant interaction between ancestor line and drug treatment (*F*_(2, 24)_ = 4.17, *p* < 0.05). Subsequent post-hoc comparisons confirmed the significant difference between F2 offspring of the paternal MIA ancestor lineage and F2 controls (*p* < 0.05; Fig. 2b) in terms of the mean distance moved after Amph treatment. There were no significant group differences with regards to spontaneous or Sal-induced locomotor activity in the F2 generation. Together, these findings demonstrate that MIA modifies Amph sensitivity in the second generation via the paternal, but not maternal, lineage without altering basal locomotor activity.

In a next step, we examined whether the transgenerational modification of Amph sensitivity induced by MIA persists into the third (F3) generation. For this purpose, we generated F3 control offspring and F3 offspring of MIA-exposed ancestors deriving from the paternal lineage as illustrated in Fig. 1b. Consistent with the effects in the second generation, F3 offspring of the paternal MIA ancestor lineage displayed a decrease in Amph-induced locomotor hyperactivity when compared to F3 controls (Fig. 2c). ANOVA of distance moved in the open field revealed a significant interaction between ancestor line and drug treatment (*F*_(1, 28)_ = 5.43, *p* < 0.05); and subsequent post-hoc comparisons confirmed the significant difference between F3 offspring of the paternal MIA ancestor lineage and F3 controls (*p* < 0.01) in terms of the mean distance moved after Amph treatment (Fig. 2c). There were no significant group differences with regards to spontaneous or Sal-induced locomotor activity in the F3 generation. Together, these findings demonstrate that transgenerational modification of Amph sensitivity induced by MIA persists into the third generation of offspring.

### Transgenerational modification of dopaminergic gene expression in the midbrain

To identify possible molecular correlates of the transgenerationally modified sensitivity to Amph, we assessed the mRNA levels of key genes for DA functioning, namely *Th* and *Nurr1*, in the adult vMB of F1, F2, and F3 offspring of MIA-exposed ancestors and corresponding controls. For F2 and F3 offspring, we included generations that were derived from the paternal lineage of MIA-exposed ancestors (as illustrated in Fig. 1). We focused on transcriptional changes in the vMB as it contains the majority of dopaminergic cell bodies in the mammalian brain [29].

**Fig. 3:**
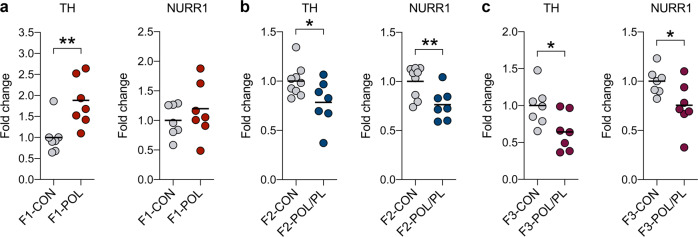
Effects of maternal immune activation on the expression of tyrosine hydroxylase (*Th*) and nuclear receptor-related 1 protein (*Nurr1*) in the ventral midbrain of male F1, F2 and F3 offspring. The graphs depict the normalized levels of *Th* and *Nurr1* mRNA levels (fold changes) of individual animals measured by quantitative RT-PCR. **a** *Th* and *Nurr1* mRNA levels in F1 offspring of control (CON) and poly(I:C)-treated (POL) mothers. **b** *Th* and *Nurr1* mRNA levels in F2 CON offspring and F2 POL offspring generated via the paternal lineage (POL/PL). **c** *Th* and *Nurr1* mRNA levels in F3 CON offspring and F3 POL offspring generated via the paternal lineage (POL/PL). *p < 0.05 and **p < 0.01, based on independent Student’s *t* tests (two-tailed).

As depicted in Fig. 3a, *Th* mRNA levels were significantly increased in F1 offspring born to poly(I:C)-treated mothers relative to F1 controls (*t*_(12)_ = 3.04, *p* < 0.01), whilst there were no significant group differences in terms of *Nurr1* mRNA levels in F1 offspring. On the other hand, F2 (Fig. 3b) and F3 (Fig. 3c) offspring of the paternal MIA ancestor lineage displayed significantly reduced mRNA levels of *Th* (F2: *t*_(14)_ = 2.23, *p* < 0.05; F3: *t*_(12)_ = 2.42, *p* < 0.05) and *Nurr1* (F2: *t*_(14)_ = 2.98, *p* < 0.01; F3: *t*_(12)_ = 2.21, *p* < 0.05) compared with corresponding control offspring. These findings thus demonstrate that MIA modifies the expression levels of DA-related genes across multiple generations, with opposing effects in the direct descants (F1 generation) and their progeny (F2 and F3 generation).

### Transgenerational effects on DNA methylation at *Th* and *Nurr1* promoter regions

We further explored whether the transgenerational modification of *Th* and *Nurr1* expression induced by MIA (Fig. 3) would be accompanied by altered DNA methylation in brain and sperm tissue. To this end, we measured DNA methylation levels of individual CpGs within the promoter region of *Th* and *Nurr1* in the vMB of MIA-exposed F1 and F2 offspring and corresponding controls, and in the sperm of F1 offspring of MIA-exposed mothers and F1 controls.

**Fig. 4:**
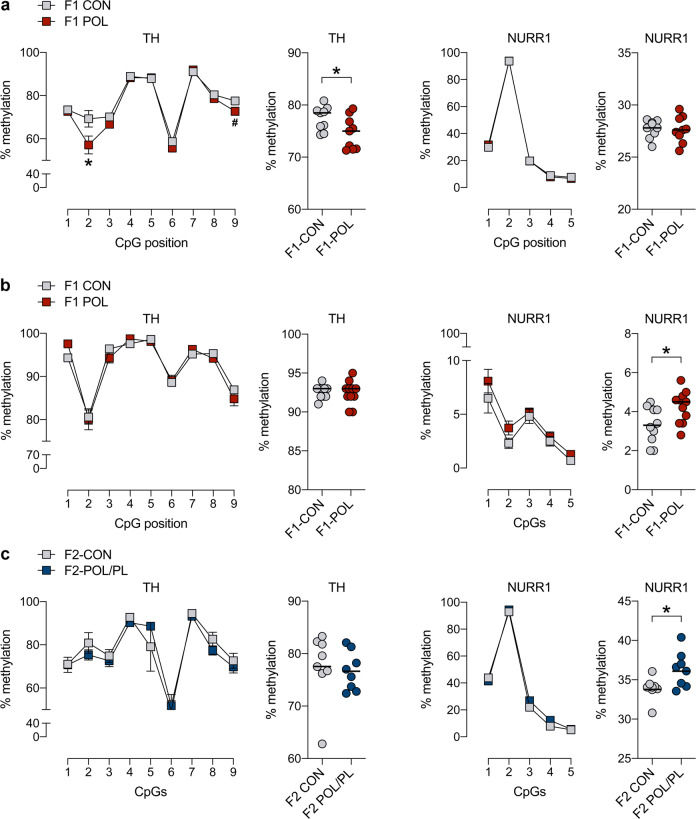
Effects of maternal immune activation on DNA methylation at promoter regions of tyrosine hydroxylase (*Th*) and nuclear receptor-related 1 protein (*Nurr1*) in the ventral midbrain (vMB) and sperm cells of male offspring. The line plots depict percent DNA methylation of specific CpGs in selected amplicons, and the scatter plots show the mean percent DNA methylation across all individual CpGs included in the corresponding amplicon. Amplicon and primer sequences and position of CpGs in each amplicon are provided in Supplementary Table S2 and S3. **a** DNA methylation levels of *Th* and *Nurr1* in the vMB of F1 offspring of control (CON) and poly(I:C)-treated (POL) mothers. *p < 0.05 and ^#^*p* = 0.059, based on ANOVA and post-hoc tests. **b** DNA methylation levels of *Th* and *Nurr1* in the sperm of F1 CON and POL offspring. *p < 0.05, based on ANOVA. **c** DNA methylation levels of *Th* and *Nurr1* in the vMB of F2 CON offspring and F2 POL offspring generated via the paternal lineage (POL/PL). *p < 0.05, based on ANOVA.

DNA at the promoter region of *Th* was generally hypomethylated in the vMB of F1 offspring born to MIA-exposed mothers relative to F1 controls (main effect of prenatal treatment: *F*_(1,16)_ = 4.64, *p* < 0.05). This effect was mainly driven by reductions in DNA methylation at CpG position 2 and, to a lesser extent, CpG position 9 in the selected amplicon (Fig. 4a), as reflected by the significant interaction of prenatal treatment and CpG position (*F*(_8,128)_ = 3.35, *p* < 0.01). Subsequent post-hoc comparisons confirmed the significant decrease in *Th* DNA methylation at CpG position 2 (*p* < 0.05) in MIA-exposed F1 compared to F1 controls and revealed a statistical trend level (*p* = 0.059) for *Th* DNA methylation at CpG position 9 (Fig. 4a). DNA methylation levels at the promoter region of *Nurr1* were not affected by MIA in the vMB of F1 offspring (Fig. 4a).

By contrast, MIA significantly affected DNA methylation of *Nurr1*, but not *Th*, in the sperm of F1 offspring (Fig. 4b). For the former gene, ANOVA revealed a significant main effect of prenatal treatment (*F*_(1,19)_ = 5.13, *p* < 0.05), while for the latter gene, there were no significant main effects or interactions involving the between-subjects factor of prenatal treatment.

The effects of MIA on sperm DNA methylation of *Nurr1*, as observed in F1 offspring of MIA-exposed mothers, were mirrored in the vMB of F2 offspring of the paternal MIA ancestor lineage (Fig. 4c). Compared to F2 controls, F2 offspring of the paternal MIA ancestor lineage displayed a small but significant (main effect of ancestor line: *F*_(1,14)_ = 4.88, *p* < 0.05) increase in DNA methylation at the *Nurr1* promoter region in the vMB. Consistent with the effects observed in F1 sperm, there were no significant group effects or interactions involving the between-subjects factor of ancestor line in the analysis of DNA methylation of *Th* in the F2 vMB (Fig. 4c).

## Discussion

The present study provides the first experimental evidence for transgenerational effects of MIA on the central DA system. Using the poly(I:C)-based MIA model in mice, our data demonstrate that behavioral and transcriptional indices of DA functions are not only altered in MIA-exposed F1 offspring, but also in their progeny. Most intriguingly, however, the direction of changes was diametrically opposite in the direct descendants of poly(I:C)-treated mothers and in subsequent generations. Compared to F1 control offspring, MIA-exposed F1 offspring displayed a potentiated sensitivity to the locomotor-stimulating effects of Amph and increased *Th* expression in the vMB. These changes are consistent with numerous previous findings in rodent [15–20] and non-human primate [21] models of MIA and are reminiscent of a hyperdopaminergic state, which in turn is a pathological hallmark of (acute) psychosis in patients with schizophrenia and related disorders [12, 39]. In stark contrast, F2 and F3 offspring of immune-challenged ancestors displayed signs of hypodopaminergia, as indicated by the presence of blunted locomotor responses to Amph and reduced expression of *Th* and *Nurr1* in the vMB. Together, these findings identify a novel role of MIA in modifying DA functions across multiple generations.

The transgenerational transmission of MIA-induced changes in Amph sensitivity occurred via the paternal lineage and was paralleled by epigenetic modifications in sperm DNA methylation. The paternal mode of the transgenerational modification of DA functions is consistent with our previous study [9], which identified a critical role of the paternal lineage in mediating non-genetic inheritance of social and fear-related abnormalities after MIA. The paternal mode of transmission is also consistent with other models of early-life adversities, such as prenatal or neonatal stress [40]. Hence, there is increasing evidence to suggest that stable epigenetic modifications in sperm cells may represent a mechanism by which exposure to environmental adversities, including immune challenges [40], can induce pathological effects across generations. In addition to our findings presented here, experimental support for the latter was also found in a recent study showing that pathogenic infection with *T. gondii* alters small-RNA profiles in sperm and induces transgenerational effects on behavior [27].

Our previous transgenerational study in the poly(I:C)-based MIA model further demonstrated that the precise nature of behavioral anomalies can differ from one generation to the next [9]. For example, we found sociability and fear-related behaviors to be affected similarly in MIA-exposed F1 offspring and their F2 and F3 progeny, whereas behavioral despair, as assessed using the Porsolt forced swim test, was found to emerge only in the F2 and F3 offspring of immune-challenged ancestors, but not in the direct descendants (F1) born to poly(I:C)-treated mothers [9]. Based on experimental work in rodent models of chronic stress exposure [11], it has been suggested that “silent molecular carriers” may cause patterns of non-genetic transgenerational inheritance that are characterized by the emergence of novel phenotypes across generations. According to this hypothesis, the exposure itself does not cause overt effects on phenotype-relevant somatic tissues in F1 offspring, but is still able to reprogram primordial germ cells of the F1 generation. The latter effect may then affect developmental processes following fertilization of the oocyte and precipitate the appearance of a certain phenotype in subsequent generations, even if it was not present in the F1 generation [11].

Notably, the data presented here suggest that some of the transgenerational effects of MIA can be modified to take opposite directions of abnormalities (e.g., from hyper- to hypodopaminergia). Besides potentiated Amph sensitivity, the spectrum of behavioral and neurochemical phenotypes emerging in F1 offspring of poly(I:C)-treated mothers involves other psychosis-related dysfunctions, including disruption of sensorimotor gating [9, 16], deficits in selective attention [15, 16] and elevations in central DA levels [41]. On the other hand, the behavioral phenotypes emerging in F2- and F3-generation offspring of MIA-exposed ancestors, as identified here and before [9], rather recapitulate disorders that are characterized by downregulated DA activity, such as major depressive disorders [14]. Currently, a causal explanation for this dichotomy is lacking. On speculative grounds, however, the contrarian directionality of effects in F1-generation versus F2- and F3-generation offspring may be related to differential and generation-dependent epigenetic effects of MIA in somatic tissue (e.g., brain) and germ cells (e.g., sperm) [11]. Here, we found that MIA increased DNA methylation of the *Nurr1* promoter in the sperm of F1 offspring, demonstrating that this early life adversity has the capacity of inducing epigenetic modifications in germ lines. A similar pattern of *Nurr1* hypermethylation was observed in the F2 generation of MIA-exposed ancestors, whereas DNA of the *Nurr1* promoter was not differentially methylated by MIA in the F1 generation. Moreover, *Nurr1* hypermethylation in the vMB of F2 MIA offspring was accompanied by reduced *Nurr1* mRNA levels in this brain region. Together, these generation-dependent effects are consistent with the notion that a certain environmental exposure such as MIA is still able to reprogram primordial germ cells of the F1 generation (and cause corresponding transcriptional changes in subsequent generations) even if it did not lead to the same changes in somatic cells of F1 offspring [11].

In keeping with the critical importance of *Nurr1* in DA cell development and functions [31–33], we also deem impaired *Nurr1* transcription to be a contributing factor for precipitation of hypodopaminergia in the F2 generation of MIA-exposed ancestors. Indeed, *Nurr1* is an obligatory transcription factor for midbrain DA cell development [31] and exerts a number of molecular functions in post-mitotic midbrain DA neurons, including the regulation of *Th* expression [32, 33]. Accordingly, reduced expression of *Nurr1* in post-mitotic DA neurons, be it induced by genetic [32, 33, 42] or environmental [43–45] manipulations, is typically associated with blunted *Th* expression. Our study is consistent with these findings and revealed a similar association between reduced *Nurr1* and *Th* expression.

We further found that MIA can alter DNA methylation of the *Th* promoter, albeit this effect was restricted to the vMB of MIA-exposed F1 offspring. The effect of MIA on *Th* DNA methylation in F1 MIA offspring extends previous investigations in poly(I:C)-based rodent models of MIA, which have identified altered DNA methylation patterns in other brain regions, including prefrontal cortex [46, 47], hypothalamus [48] and striatum [49]. Interestingly, genome-wide profiling of DNA methylation in F1 offspring born to poly(I:C)-treated mice suggests that exposure to this environmental factor leads to a global overrepresentation of DNA hypomethylation in somatic tissue such as the prefrontal cortex [47] and hypothalamus [48]. The present data showing reduced *Th* DNA methylation in the vMB of MIA-exposed F1 offspring is consistent with these previous reports and suggest that MIA has the capacity to reduce DNA methylation in somatic cells of the midbrain regions as well. Reduced *Th* promoter DNA methylation was further associated with increased *Th* expression in the vMB, which in turn is in line with the permissive transcriptional effects of DNA hypomethylation in gene promoter regions [50, 51]. Additional investigations will be required, however, to test whether hypomethylation of the *Th* promoter is causally linked with increased *Th* expression in the vMB of F1 offspring born to MIA-exposed mothers.

We acknowledge a number of other limitations in our study. First, our molecular analyses only included two specific genes relevant for DA development and functions, namely *Th* and *Nurr1*. Arguably, the molecular machinery pertaining to DA signaling involves an intricate network of genes [52, 53], and therefore, an extension to other gene sets would be desirable to further examine the specificity of our findings. A second limitation of our study is that we did not include female mice in the behavioral studies of F2- and F3-generation offspring, whereas both sexes were included in the F1 generation. Hence, it remains to be explored whether MIA induces similar transgenerational effects on Amph sensitivity and DA-related molecular profiles in males and females. An extension of our investigations to the female sex also appears crucial in view of the fact that distinct molecular mechanisms may drive the emergence of similar phenotypes in males and females [54]. Third, the postulated involvement of epigenetic modifications in sperm DNA, which may contribute to the modification of DA functions in the F2 generation of immune-challenged ancestors, still awaits verification through additional analyses. While our study focused on DNA methylation in promoter regions, there are numerous other epigenetic processes that may contribute to the transgenerational effects of MIA [5, 6], including altered expression of non-coding RNAs such microRNAs [55, 56]. Likewise, the germline dependence of the MIA-induced transgenerational effects on DA functions should be explored further. While paternal experience can influence offspring development via germline inheritance, mothers can also serve as a modulating factor in determining the impact of paternal influences on offspring development [40]. The lack of overt changes in maternal behavior (Supplementary Fig. S1), however, underscores the importance of male gametes underlying the transgenerational effects of MIA in our study. The germ-line dependence of MIA-induced changes should be further ascertained using experimental methods that can establish mechanistic causality. Artificial insemination [57, 58], for example, may be used to test the hypothesis that the modification of DA functions in the F2 generation of immune-challenged ancestors involves epigenetic modifications of sperm cells.

Despite these limitations, we conclude that MIA has the potential to modify DA functions across generations, at least within well-defined experimental settings. The broader relevance of these data is that they show that the transgenerational modification of a DA-related functional readout after MIA is mirrored by a transgenerational modification of molecular components of the central DA system, highlighting a remarkable correspondence of the two domains. To the best of our knowledge, our study is the first demonstration of altered sperm DNA methylation after MIA. Our findings thus suggest that MIA can induce epigenetic modifications in both somatic and (male) germ cells, which in turn may point to a potential mechanism by which MIA can induce pathologies in multiple generations even in the absence of additional immune exposures. Further studies on the identification of epigenetic and transgenerational effects in MIA-induced neurodevelopmental disorders may help to identify complex patterns of transgenerational disease transmission beyond genetic inheritance.

## Funding and disclosure

The authors declare no conflict of interest. The present study was supported by the Swiss National Science Foundation (Grant No. 310030-188524) awarded to UM and by a grant from the Olga-Mayenfisch-Foundation awarded to UWS. Open access funding provided by Universität Zürich.

## Supplementary information

Supplementary Table S1

Supplementary Information
