# Supplementary material for: Transgenerational modification of dopaminergic dysfunctions induced by maternal immune activation
Source: Neuropsychopharmacology. 2020 Sep 12;46(2):404–12. doi: 10.1038/s41386-020-00855-w (PMC7852665; doi:10.1038/s41386-020-00855-w)
Supplement: Supplementary file 2 — Supplementary Information [file 41386_2020_855_MOESM2_ESM.pdf]

SUPPLEMENTARY INFORMATION

Transgenerational modification of dopaminergic dysfunctions induced by maternal immune activation

Ulrike Weber-Stadlbauer[1*], Juliet Richetto[1,2], Ramona A. J. Zwamborn[3,4], Roderick C Slieker[4,5], Urs Meyer[1,2*]

[1]Institute of Pharmacology and Toxicology, University of Zurich-Vetsuisse, Zurich, Switzerland
[2]Neuroscience Center Zurich, University of Zurich and ETH Zurich, Zurich, Switzerland
[3] Department of Neurology, UMC Utrecht Brain Center, Utrecht, The Netherlands
[4] Department of Biomedical Data Sciences, Section Molecular Epidemiology, Leiden, The Netherlands
[5] Department of Cell and Chemical Biology, Leiden University Medical Center, Leiden, The Netherlands

Correspondence:

Ulrike Weber-Stadlbauer, Ph.D.
Institute of Pharmacology and Toxicology
University of Zurich-Vetsuisse
Winterthurerstrasse 260,
8057 Zurich,
Switzerland
E-mail: ulrike.weber@uzh.ch
Tel.: +41 44 635 87 77; Fax.: +41 44 635 89 10

SUPPLEMENTARY MATERIALS AND METHODS

Animals

C57BL6/N mice, originally obtained from Charles River (Sulzfeld, Germany), were kept in our in-house specific pathogen-free facility until breeding began to generate poly(I:C) and control offspring (see below). All animal breeding and holding rooms were temperature- and humidity-controlled (21 ± 1 °C, 55 ± 5%) and kept under a reversed light-cycle (lights off: 7:00 A.M. to 7:00 P.M.). All animals had *ad libitum* access to food (Kliba 3436, Kaiseraugst, Switzerland) and water throughout the entire study. All procedures described in the present study had been previously approved by the Cantonal Veterinarian's Office of Zurich, and all efforts were made to minimize the number of animals used and their suffering.

Timed mating procedures

To obtain timed-pregnant F0 dams for the subsequent maternal manipulations (see below), we used a timed-mating procedure as established and validated before [1]. Each mating cage contained 2 females and 1 male animal. Before mating, they were kept in a partitioned cage for 2 days, which allowed olfactory but not physical contact between male and female animals. On the third day of partitioning, female and male animals were brought together and allowed to mate. Successful mating was verified on the next morning by the presence of a vaginal plug, and the day was referred to as gestational day (GD) 0. Upon successful mating, the male was removed from the cage, and females were placed into a new cage, so that the females were kept alone throughout the entire gestation and post-partum rearing periods. The same timed-mating procedure was used for the generation of F2 and F3 offspring, with the exception that timed-pregnant F1 and F2 dams did not receive any treatments.

Maternal immune activation in F0 mothers

Pregnant F0 dams on GD 9 were randomly assigned to receiving either a single injection of poly(I:C) (potassium salt, P9582, lot number: 086M4045; Sigma–Aldrich, Buchs, St. Gallen,

Switzerland) or vehicle. Poly(I:C) (5 mg/kg) was dissolved in sterile pyrogen-free 0.9% NaCl (vehicle) solution to yield a final concentration of 1 mg/ml and was administered intravenously (i.v.) into the tail vein under mild physical constraint as described before [1, 2]. The dose of poly(I:C) was selected based on our previous dose-response studies [3, 4]. For all experimental series involving F0 exposures, a total of 18-24 pregnant dams were used, half of which were allocated to the poly(I:C) treatment, and the other half to the vehicle treatment. The selected gestational window (i.e., GD 9) was selected based on previous findings showing that poly(I:C) exposure on GD 9 leads to multiple behavioral abnormalities in the adult offspring, including dopaminergic dysfunctions such as hypersensitivity to systemic amphetamine treatment[1, 5]. The selected gestational window (i.e., GD 9) in mice corresponds roughly to the middle of the first trimester of human pregnancy with respect to developmental biology and percentage of gestation from mice to humans [6]. We have previously verified the effectiveness of this poly(I:C) lot and administration protocol in C57BL6/N mice in terms of the elicited cytokine-associated inflammatory response in maternal and fetal tissues [4, 7].

Assessment of maternal behavior

The assessment of maternal behavior started after birth and lasted for five days (i.e., until PND 5). We focused on this period because maternal observations over the first days postpartum are expected to be most critical for the reliable assessment of possible differences in maternal behavior [8]. Maternal care was measured every 4 h, with one scoring lasting 120 sec. Hence, there were a total of 6 120-sec recording sessions per 24 h (starting at 08.30, 12.30, 16.30. 20.30, 00.30 and 04.30).

Maternal behavior was subdivided into different categories[9]:

 Licking/grooming: The mother was licking and/or grooming the pups, clearly recognizable by the movement of her head and/or paws.

 Nursing: This refers to feeding the pups and comprised all categories of nursing, including kyphosis (nursing the pups in a dorsal upright position, with rigid fore- and/or hindlimbs) and

prone nursing (nursing the pups while lying flat on the top of the pups, with little or no limb support).

 Off nest: This was scored when the mother is somewhere in the cage without any contact to the pups.

The abovementioned categories (licking/grooming, nursing and off-nest behavior) comprised 80-90% of the maternal behavior across the first PNDs and are summarized in **Supplementary Figure S1**. We furthermore assessed other categories, including self-licking/-grooming, eating/drinking and nest-building, but found no significant group differences between in these measures (data not shown).

Allocation of F1 offspring and production of subsequent generations

All F1 offspring were weaned and sexed on postnatal day (PND) 21. Littermates of the same sex were caged separately and maintained in groups of 3 to 5 animals per cage. Upon reaching adulthood (PND 90 onwards), F1 offspring were either allocated to behavioral testing (see below) or molecular analyses and breeding, the latter of which served to produce subsequent generations of immune-challenged or control ancestors. Hence, we always used behaviorally naïve littermates for molecular analyses and as breeding pairs to obtain the F2 and F3 generations, thereby avoiding possible confounds arising from prior behavioral testing. Timed-mating procedures, as fully described above, were used to generate F2 and F3 offspring.

In one series of experiments, we dissected the maternal (ML) and paternal lineages (PL) of F1 poly(I:C) offspring for the subsequent generation of F2 offspring (**Fig. 1a**). To obtain F2 poly(I:C) offspring via the ML, we crossed female F1 poly(I:C) offspring with male F1 control offspring (N = 6 litters); and to generate F2 poly(I:C) offspring via the PL, we mated male F1 poly(I:C) offspring with female F1 control offspring (N = 7 litters). F1 control males and F1 control females were crossed to obtain the F2 control lineage (N = 7 litters) (**Fig. 1a**).

Another series of experiments was performed to generate F3 offspring with poly(I:C)-exposed or control ancestors, thereby focusing on the PL (**Fig. 1b**). To this end, F1 males born to poly(I:C)-exposed mothers were mated with F1 control females to generate PL-derived F2

poly(I:C) offspring. The latter were then mated with F2 control offspring to obtain PL-derived F3 poly(I:C) offspring (*N* = 9 litters). Control F3 offspring were generated by crossing F2 control males and F2 control females (*N* = 8 litters). **Table 1** (see main text) summarizes the number of animals used per group in each experiment.

Amphetamine sensitivity test in F1, F2 and F3 offspring

In each generation, we assessed amphetamine sensitivity by measuring locomotor activity in an open field apparatus, as described in detail previously [10, 11]. The behavioral test was conducted during the dark phase of the light-dark cycle. The test apparatus consisted of 4 identical square arenas (40 x 40 x 35 cm) made of wood and painted in grey. It was positioned in a testing room with dimly diffused lighting (30 lux as measured in the center of the open field arena). A camera, mounted above the open field, captured images and transmitted them to a PC running the Ethovision tracking system (Noldus Technology, Wageningen, Netherlands). At the beginning of a test session, the animals were placed into the center of the corresponding open field arena. Animals were first allowed to explore the arena freely for 30 minutes, which served to habituate the animals to the test apparatus and to measure basal locomotor activity. Animals were then quickly removed from the open field and received an injection of D-amphetamine sulfate (Amph, Sigma-Aldrich, Switzerland; 2.5 mg/kg, 5ml/kg, i.p.) or saline (Sal; 5ml/kg, i.p.) solution and were immediately placed back to the appropriate arena again. The locomotor reactions to the acute drug or Sal challenges were then recorded for a total of 90 minutes.

The Amph sensitivity test in F1-generation animals was replicated using an independent cohort of male and female poly(I:C)-exposed and control offspring, thereby using a within-subjects drug administration design (see **Supplemental Figure S2**).

Mouse brain collection for gene expression and DNA methylation analyses

Behaviorally naïve mice were killed by decapitation for subsequent gene expression and DNA methylation analyses. Brains were rapidly extracted from the skull (within < 30 s), immediately

frozen on powdered dry ice and kept at -80 °C until further processing. Frozen coronal sections were then prepared using razorblade cuts along the following coordinates with respect to bregma: -2.9 to -3.8 mm. Micropunches of the ventral midbrain were then generated using a micropunch needle (1 mm in diameter) as described before [12]. The punches were then immediately processed further for DNA and RNA extraction. For sperm cell collection, the intraperitoneal cavity was cut open from the sternum to the penis to expose the testes. The cauda epididymis was dissected and sliced with several cuts to rupture the tissue and release sperm cells, which were immediately transferred into an Eppendorf tube, snap-frozen and stored in -80 °C until further processing.

RNA Isolation and Real-Time PCR Analyses

Total RNA was isolated using the Qiagen AllPrep DNA and RNA Mini kit (Qiagen, Switzerland) according to the manufacturer's instructions, and quantified by spectrophotometric analysis. An aliquot of each RNA sample was then treated with DNase to avoid DNA contamination. RNA was analysed by TaqMan RT-PCR instrument (CFX384 real-time system, Bio-Rad Laboratories) using the iScript one-step RT-PCR kit for probes (Bio-Rad Laboratories). The samples were run in 384-well formats in triplicates as multiplexed reactions with a normalizing internal control (36B4). We choose 36B4 as internal standard for gene expression analyses since its expression was not affected by the prenatal treatments [13].

Thermal cycling was initiated with an incubation at 50°C for 10 min (RNA retrotranscription) and then at 95°C for 5 min (TaqMan polymerase activation). After this initial step, 39 cycles of PCR were performed. Each PCR cycle consisted of heating the samples at 95°C for 10 s to enable the melting process and then for 30 s at 60°C for the annealing and extension reaction. Relative target gene expression was calculated according to the 2(-Delta Delta C(T)) method [14]. The mouse TaqMan gene expression assays Mm00447557 and Mm00443060 were used for *Th* and *Nurr1*, respectively. 1-2 male offspring per litter were randomly selected and tested to minimize possible confounds arising from litter effects, with a sample size ranging from 7-9 per group.

DNA Isolation and assessment of DNA methylation differences by EpiTYPER

DNA from the ventral midbrain (vMB) and sperm was extracted with the Qiagen AllPrep DNA and RNA Mini kit and Qiagen DNeasy blood and tissue kit (both Qiagen, Switzerland), respectively, according to the manufacturer's instructions. To assess methylation levels at the genes of interest, specific CpGs were analyzed using *EpiTYPER*. This technique detects and quantifies DNA methylation using base-specific cleavage and matrix-assisted laser desorption/ionization time-of-flight mass spectrometry [15]. Genomic DNA was treated with bisulfite and amplified by PCR with primers specific to genomic regions of the genes of interest (*Th* and *Nurr1*): 95°C for 5 min, followed by 49 repeated cycles (95°C for 40 s, 56°C for 40 s, 72°C for 40 s) and a last step at 72°C for 5 min. Unincorporated dNTPs leftover from amplification were neutralized using shrimp alkaline phosphatase (SAP). Then, to obtain fragmented RNA molecules, in-vitro RNA transcription with subsequent base-specific cleavage using RNase A was performed. Both methylated and non-methylated regions were cleaved at every T to produce fragments that are identical in length and differ only in their nucleotide composition. The samples were then conditioned so that the products could be processed in the MALDI-TOF mass spectrometer and could be analyzed by their mass spectra. In analyzing the mass spectrum, the relative amount of methylation can be calculated by comparing the difference in signal intensity between mass signals derived from methylated and non-methylated template DNA [15].

The primer sequences used to amplify genomic regions of the genes of interest (*Th* and *Nurr1*) are shown in **Supplementary Table S2**. The primers were designed for the promoter region of the genes covering the regions with the most CpG sites. The regions of interest were determined using the genomic sequence of the DNA obtained from UCSC genome browser mm9 (http://genome.ucsc.edu/). The selected regions were based on active methylation marks (H3K4, H3K9m1, H3K27m1), their location compared to the transcription start site of the gene of interest, and CpG coverage. After determination of the region of interest, primer sequences were designed with a T7-promoter tag using SEQUENOM EpiDesigner BETA

(http://www.epidesigner.com/). **Supplementary Table S3** depicts the sequences of the selected amplicons assessed with EpiTYPER as well as the position of CpGs in each amplicon. Methylation of the numbered CpGs was accessible to quantification by *EpiTYPER* and corresponds to the CpG numbers presented in **Figure 4** of the main manuscript. 1-2 male offspring per litter were randomly selected and tested to minimize possible confounds arising from litter effects, with a sample size ranging from 8-11 per group.

Statistical Analyses

All data were analyzed by parametric analysis of variance (ANOVA) followed by Tukey's post-hoc test for multiple comparisons whenever appropriate. Individual offspring were taken as the experimental unit. Statistical analyses were performed using the statistical software StatView (version 5.0). and Prism (version 8.4) and statistical significance was set at $p < 0.05$.

In the Amph sensitivity test of the F1 offspring, the total distance moved was expressed as a function of 5-min-bins and analyzed by a 2 x 2 x 2 x 6 (prenatal treatment x sex x Amph condition x bins) repeated-measures ANOVA for the initial habituation phase, and by a 2 x 2 x 2 x 18 (prenatal treatment x sex x Amph condition x bins) repeated-measures ANOVA for the subsequent test phase. The Amph sensitivity test of second- generation offspring was analyzed by a 3 x 2 x 6 (ancestor line x Amph condition x bins) repeated-measures ANOVA for the initial habituation phase, and by a 3 x 2 x 18 (ancestor line x Amph condition x bins) repeated-measures ANOVA for the subsequent test phase. In the Amph sensitivity test of the F3 offspring, the total distance moved was expressed as a function of 5-min-bins and analyzed by a 2 x 2 x 6 (ancestor line x Amph condition x bins) repeated-measures ANOVA for the initial habituation phase, and by a 2 x 2 x 18 (ancestor line x Amph condition x bins) repeated-measures ANOVA for the subsequent test phase. All gene expression analyses were analyzed using independent student's *t* tests (two-tailed). Methylation of genes of interest was expressed as a function of individual CpGs and analyzed using a 2 x 9 and a 2 x 5 ANOVA (prenatal treatment x CpG position) for *Th* and *Nurr1*, respectively.

SUPPLEMENTARY TABLES

Amplicon	*Genomic location (NCBI37/mm9)*	Forward Primer (capital letters: gene specific)	Reverse Primer (capital letters: gene specific)
Th	Chr7: 150085886-150086174	5’-aggaagagagGAGTTTTGTTT TTATAGTTTTTGTTAGG-3’	5’-cagtaatacgactcactatagggagaaggct TAAATATCTCCTATCCCAAAACACC -3’
Nurr1	Chr2: 56976875 - 56977024	5’-aggaagagagTTTGTAGAGA AAGTTTTTTTAGATGG-3’	5’-cagtaatacgactcactatagggagaaggctAA AATTATCAATAAAAACCCCCAAA-3’

Supplementary Table S2. Genomic location and sequences of the primers used to amplify genomic regions of the genes of interest, *Th* and *Nurr1.*

Gene	Product size / assayed CpGs (bp/n)
Th	
GAGTTCTGTCTCCACAGCCCTTGCCAGGCAGG**CG**^{1}GCCTCTTAAAGGCCAGGCT<br>GA**CG**^{2}TCAAAGCCCCTCTGGGTCCCCCACCTCCTACCTCCTG**CG**^{3}CATCCTCTCC<br>A**CG**^{4}CCTGCTGTGCCTGAGGGAGG**CG**^{5}GGGGTGGGCACAGAGCGAGGG**CG**^{6}AC<br>GGACGAGAGAGGCCTC**CG**^{7}TCCCATTAGATCTAATTGCATCCACTGT**CG**^{8}CAGGC<br>ACCTGCCTCTGAATCACCCTC**CG**^{9}CCCTAGACACGACATGAAGACAGGGGCTGG<br>CTGGTGTTCTGGGACAGGAGACATCTA	**289/9**
Nurr1	
CCTGCAGAGAAAGCCCCTTTAGATGGCAGCCTTGACTGGACTTCCAA**CG**^{1}GCCA<br>AAGGACCAGACTTGGAAGATCACAACAGGTTG**CG**^{2}CCATCCTGAAGGCCACAAA<br>GATGTAAAGAAAGGACAGTGGGGCTGAGATGGTGAGTCCTGGAACCCAGGCCT<br>**CG**^{3}CTGCTTCTTCTGGACCCT**CG**^{4}GGGGGCACAGTGGCTTAAAAGTAGCT**CG**^{5}GG<br>CAGACATTTAAGACGCCAAGTTCTTCTTTATTTGGGGGTTTTCACTGACAATTTCC	**266/5**

Supplementary Table S3. Sequences of the selected amplicons assessed with EpiTYPER and position of CpGs in each amplicon. Methylation of the numbered CpGs (in bold font) was accessible to quantification by EpiTYPER and corresponds to the CpG numbers presented in **Fig. 4**. The underlined CpGs were not measurable for technical reasons.

Experiment	Generation	Dependent Measures	ANOVA	Significant Effects	DF	F-Value	P-Value	Figure
**Amph sensitivity**	F1	(§) Total distance moved	2 × 2 × 2 × 18 (prenatal treatment x drug treatment x sex x bins)	Prenatal treatment x drug treatment	(1,37)	4.44	**<0.05**	Fig. 2a
		(§) Total distance moved following Amph	2 × 2 × 18 (prenatal treatment × sex × bins)	Prenatal treatment	(1,19)	4.85	**<0.05**	
	F2	(§) Total distance moved	3 × 2 × 18 (ancestor line × drug treatment × bins)	Ancestor line x drug treatment	(2,24)	4.17	**<0.05**	Fig. 2b
		(§) Total distance moved following Amph	3 × 18 (ancestor line × bins)	Ancestor line	(2,12)	4.18	**<0.05**	
	F3	Total distance moved	2 × 2 × 18 (ancestor line × drug treatment × bins)	Ancestor line x drug treatment	(1,27)	4.21	**<0.05**	Fig. 2c
		Total distance moved following Amph	2 × 18 (ancestor line × bins)	Ancestor line	(1,15)	5.49	**<0.05**	

Supplementary Table S4. Secondary analyses of amphetamine sensitivity, for which the litter (i.e. treated mothers) were used as the experimental unit. The table specifies the relevant dependent measures and summarizes the significant main effects of and interactions between independent factors. The table also specifies the corresponding degrees of freedom (DF) and F-values. Note: In analyses denoted with the symbol (§), the number of litters is equivalent to the number of offspring. Hence, in these analyses, only 1 offspring per litter and sex were used, and consequently, the outcomes of the primary (see main text) and secondary analyses are identical.

Experiment	Generation	Dependent Measures	Test	DF	t-Value	P-Value	Figure
**Gene Expression *Th***	F1	(§) mRNA relative expression (fold change)	Two-tailed t-test	12	3.04	**<0.01**	Fig. 3a
	F2	mRNA relative expression (fold change)	Two-tailed t-test	12	2.08	**<0.05**	Fig. 3b
	F3	mRNA relative expression (fold change)	Two-tailed t-test	10	2.38	**<0.05**	Fig. 3c
**Gene Expression *Nurr1***	F1	(§) mRNA relative expression (fold change)	Two-tailed t-test	12	0.89	0.39	Fig. 3a
	F2	mRNA relative expression (fold change)	Two-tailed t-test	12	2.84	**<0.05**	Fig. 3b
	F3	mRNA relative expression (fold change)	Two-tailed t-test	10	2.34	**<0.05**	Fig. 3c

Supplementary Table S5. Secondary analyses of gene expression, for which the litter (i.e. treated mothers) were used as the experimental unit. The table specifies the dependent measures for each test and summarizes the main effects between groups. The table also specifies the corresponding degrees of freedom (DF) and t-values. Note: In analyses denoted with the symbol (§), the number of litters is equivalent to the number of offspring. Hence, in these analyses, only 1 offspring per litter and sex were used, and consequently, the outcomes of the primary (see main text) and secondary analyses are identical.

Experiment	Generation	Dependent Measures	ANOVA	Effects	DF	F-Value	P-Value	Figure
**DNA methylation *Th***	F1 Brain	(§) Relative DNA methylation (%)	2 × 9 (prenatal treatment × CpGs)	Prenatal treatment	(1,16)	4.64	**<0.05**	Fig. 4a
				Prenatal treatment × CpG	(8,128)	3.35	**<0.01**	
	F1 Sperm	Relative DNA methylation (%)	2 × 9 (prenatal treatment × CpGs)	Prenatal treatment	(1,18)	0.03	0.87	Fig. 4b
				Prenatal treatment × CpG	(8,144)	1.45	0.19	
	F2 Brain	Relative DNA methylation (%)	2 × 9 (prenatal treatment × CpGs)	Prenatal treatment	(1,12)	4.21	0.06	Fig. 4c
				Prenatal treatment × CpG	(8,96)	1.01	0.43	
**DNA methylation *Nurr1***	F1 Brain	(§) Relative DNA methylation (%)	2 × 5 (prenatal treatment × CpGs)	Prenatal treatment	(1,16)	0.1	0.76	Fig. 4a
				Prenatal treatment × CpG	(4,64)	0.19	0.94	
	F1 Sperm	Relative DNA methylation (%)	2 × 5 (prenatal treatment × CpGs)	Prenatal treatment	(1,18)	4.94	**<0.05**	Fig. 4b
				Prenatal treatment × CpG	(4,72)	0.47	0.75	
	F2 Brain	Relative DNA methylation (%)	2 × 5 (prenatal treatment × CpGs)	Prenatal treatment	(1,12)	6.88	**<0.05**	Fig. 4c
				Prenatal treatment × CpG	(4,48)	1.74	0.16	

Supplementary Table S6. Secondary analyses of DNA methylation at promotor regions, for which the litter (i.e. treated mothers) were used as the experimental unit. The table specifies the dependent measures for each test and summarizes the main effects of and interactions between independent factors. The table also specifies the corresponding degrees of freedom (DF) and F-values. Note: In analyses denoted with the symbol (§), the number of litters is equivalent to the number of offspring. Hence, in these analyses, only 1 offspring per litter and sex were used, and consequently, the outcomes of the primary (see main text) and secondary analyses are identical.

SUPPLEMENTARY FIGURES

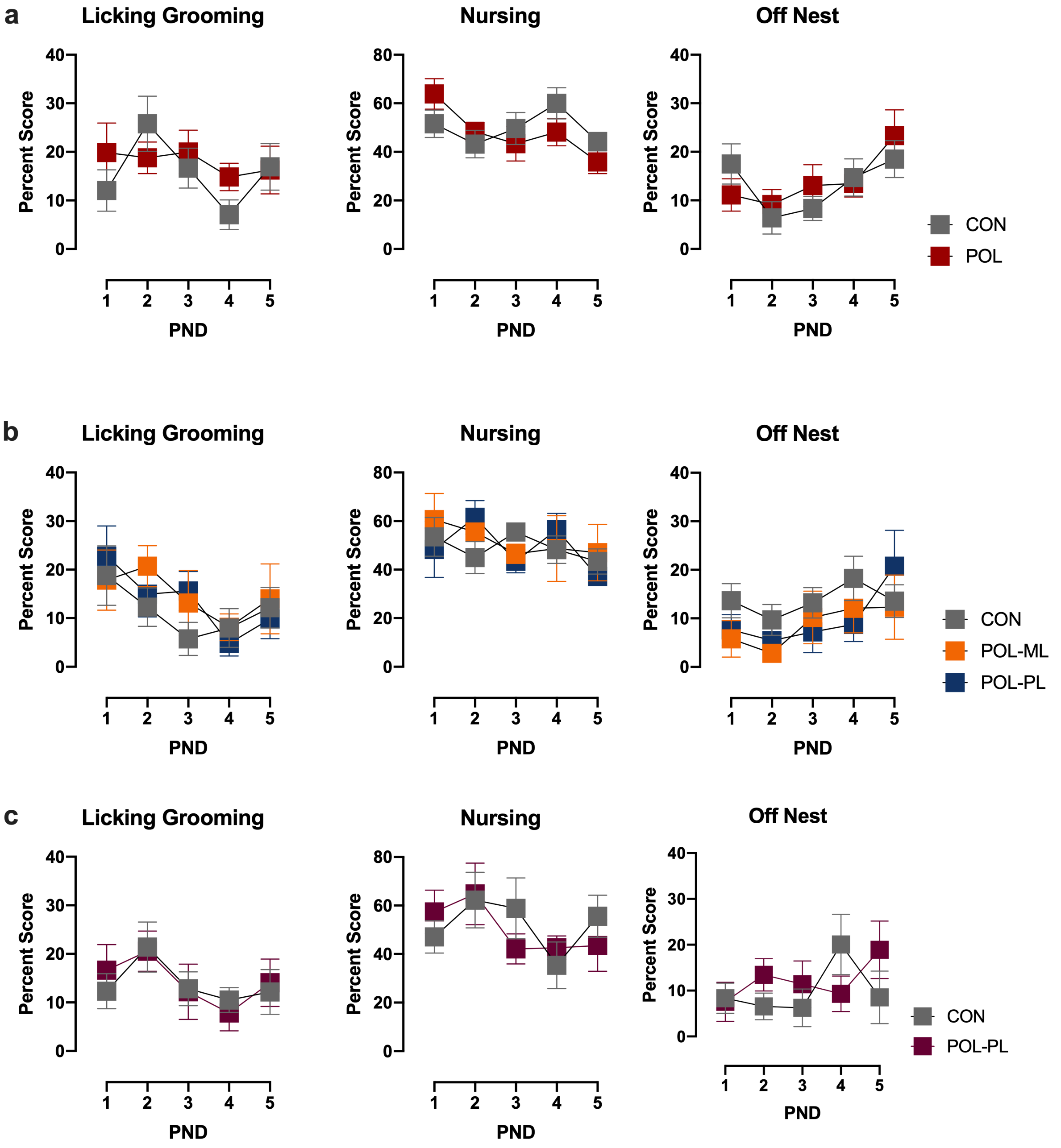

Supplementary Figure S1. Effects of maternal immune activation on postpartum maternal behavior in F1, F2 and F3 mothers. Maternal behavior was assessed on postnatal days 1-5. Each individual maternal behavior score is expressed as a proportion (in percent) of the total scores, and is depicted as a function of postnatal days (line plot). (**a**) Maternal behavior scores in control (CON) and poly(I:C)-treated (POL) mothers of F1 offspring. *N(CON)=10, N(POL)=11.* (**b**) Maternal behavior scores in mothers of F2 offspring generating the maternal (POL-ML) and paternal (POL-PL) lineages. *N(CON)= 7, N(POL-ML)=6, N(POL-PL)=7.*(**c**) Maternal behavior scores in mothers of F3 offspring following the paternal (POL-PL) lineage. *N(CON)=8, N(POL-PL)=9.* All values are means ± SEM.

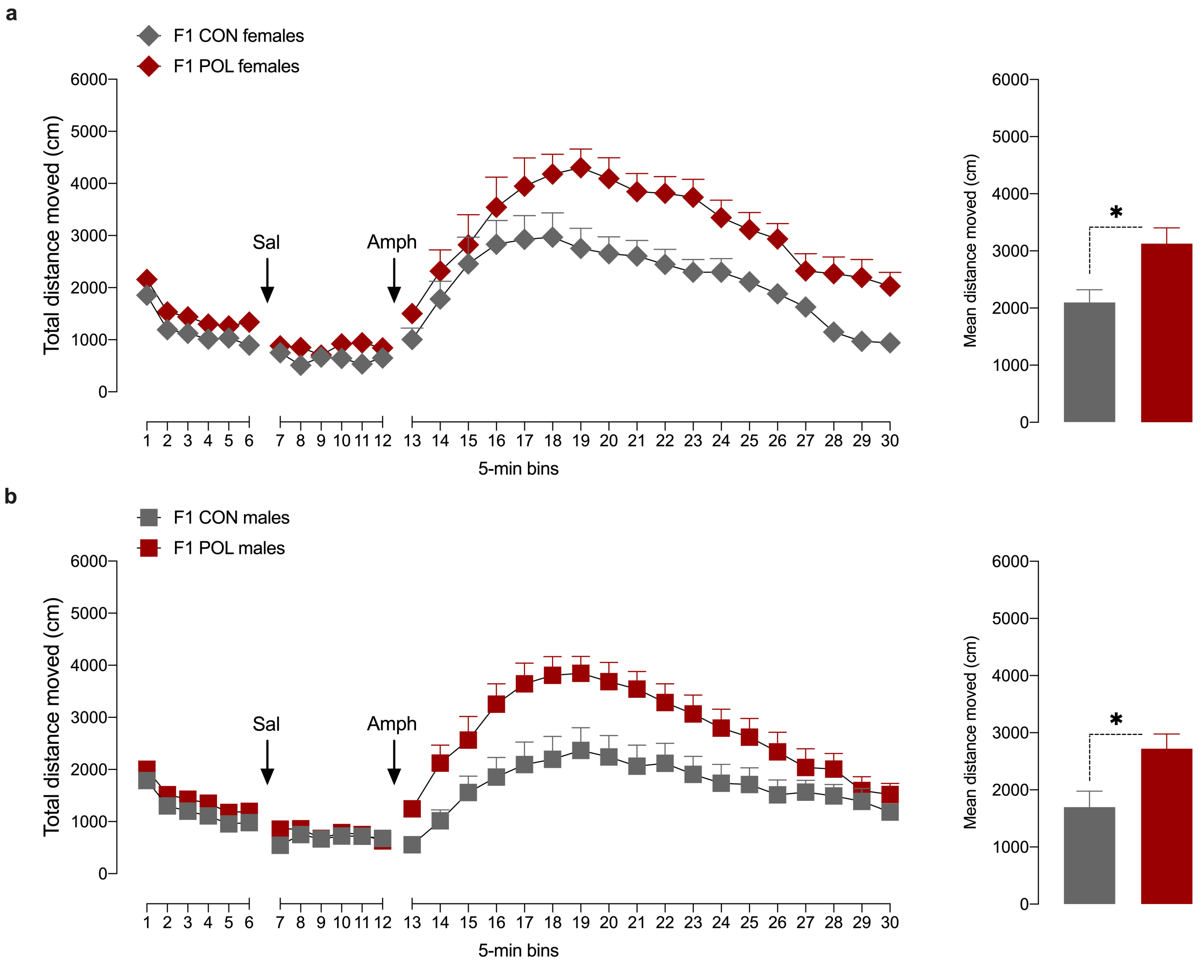

Supplementary Figure S2. Effects of maternal immune activation on the locomotor reaction to acute systemic amphetamine challenge in F1 female and male offspring. The line plots show the distance moved (cm) per 5-minute bins during the initial acclimatization phase and subsequent saline (Sal) and amphetamine (Amph) treatment phases; the bar plots depict the mean distance moved during the amphetamine phase. (**a**) Locomotor activity levels in female F1 offspring of control (CON) and poly(I:C)-treated (POL) mothers. *N (CON) = 9, N(POL) = 9.* $F_{(1,16)}$ = 8.51, *p < 0.05. (**b**) Locomotor activity levels in male offspring of control (CON) and poly(I:C)-treated (POL) mothers. *N(CON) = 10, N(POL) = 10.* $F_{(1,18)}$ = 7.34, *p < 0.05. All values are means + SEM.

SUPPLEMENTARY REFERENCES

1. Weber-Stadlbauer, U., Richetto, J., Labouesse, M.A., Bohacek, J., Mansuy, I.M., and Meyer, U., *Transgenerational transmission and modification of pathological traits induced by prenatal immune activation.* Mol Psychiatry, 2017. **22**(1): p. 102-112.
2. Meyer, U., Nyffeler, M., Engler, A., Urwyler, A., Schedlowski, M., Knuesel, I., et al., *The time of prenatal immune challenge determines the specificity of inflammation-mediated brain and behavioral pathology.* J Neurosci, 2006. **26**(18): p. 4752-62.
3. Meyer, U., Feldon, J., Schedlowski, M., and Yee, B.K., *Towards an immuno-precipitated neurodevelopmental animal model of schizophrenia.* Neurosci Biobehav Rev, 2005. **29**(6): p. 913-47.
4. Mueller, F.S., Polesel, M., Richetto, J., Meyer, U., and Weber-Stadlbauer, U., *Mouse models of maternal immune activation: Mind your caging system!* Brain Behav Immun, 2018.
5. Vuillermot, S., Weber, L., Feldon, J., and Meyer, U., *A longitudinal examination of the neurodevelopmental impact of prenatal immune activation in mice reveals primary defects in dopaminergic development relevant to schizophrenia.* J Neurosci, 2010. **30**(4): p. 1270-87.
6. Clancy, B., Darlington, R.B., and Finlay, B.L., *Translating developmental time across mammalian species.* Neuroscience, 2001. **105**(1): p. 7-17.
7. Mueller, F.S., Richetto, J., Hayes, L.N., Zambon, A., Pollak, D.D., Sawa, A., et al., *Influence of poly(I:C) variability on thermoregulation, immune responses and pregnancy outcomes in mouse models of maternal immune activation.* Brain Behav Immun, 2019. **80**: p. 406-418.
8. Champagne, F.A., Francis, D.D., Mar, A., and Meaney, M.J., *Variations in maternal care in the rat as a mediating influence for the effects of environment on development.* Physiol Behav, 2003. **79**(3): p. 359-71.
9. Schwendener, S., Meyer, U., and Feldon, J., *Deficient maternal care resulting from immunological stress during pregnancy is associated with a sex-dependent enhancement of conditioned fear in the offspring.* J Neurodev Disord, 2009. **1**(1): p. 15-32.
10. Bitanihirwe, B.K., Peleg-Raibstein, D., Mouttet, F., Feldon, J., and Meyer, U., *Late prenatal immune activation in mice leads to behavioral and neurochemical abnormalities relevant to the negative symptoms of schizophrenia.* Neuropsychopharmacology, 2010. **35**(12): p. 2462-78.
11. Stadlbauer, U., Weber, E., Langhans, W., and Meyer, U., *The Y2 receptor agonist PYY(3-36) increases the behavioural response to novelty and acute dopaminergic drug challenge in mice.* Int J Neuropsychopharmacol, 2014. **17**(3): p. 407-19.
12. Purves-Tyson, T.D., Weber-Stadlbauer, U., Richetto, J., Rothmond, D.A., Labouesse, M.A., Polesel, M., et al., *Increased levels of midbrain immune-related transcripts in schizophrenia and in murine offspring after maternal immune activation.* Mol Psychiatry, 2019.
13. Richetto, J., Labouesse, M.A., Poe, M.M., Cook, J.M., Grace, A.A., Riva, M.A., et al., *Behavioral effects of the benzodiazepine-positive allosteric modulator SH-053-2'F-S-CH(3) in an immune-mediated neurodevelopmental disruption model.* Int J Neuropsychopharmacol, 2015. **18**(4).
14. Livak, K.J. and Schmittgen, T.D., *Analysis of relative gene expression data using real-time quantitative PCR and the 2(-Delta Delta C(T)) Method.* Methods, 2001. **25**(4): p. 402-8.
15. Suchiman, H.E., Slieker, R.C., Kremer, D., Slagboom, P.E., Heijmans, B.T., and Tobi, E.W., *Design, measurement and processing of region-specific DNA methylation assays: the mass spectrometry-based method EpiTYPER.* Front Genet, 2015. **6**: p. 287.
